# Some Thoughts: Scattered Hair and There!

**DOI:** 10.4103/0974-7753.51921

**Published:** 2009

**Authors:** Shyam B Verma

**Affiliations:** Consultant Dermatologist Gujarat, Hon. Dermatologist to H.E. The Governor of Gujarat State, India/Clinical Associate Professor, University of Virginia, Charlottesville/Clinical Assistant Professor, SUNY at Stony Brook, USA

It is only fitting that this journal has been born in India, a country which has given so much attention to hair and its esthetics from times immemorial. A country which has attached so much importance to hair in mythology, which has witnessed various styles of hair evolved over millennia, which has put so much importance to its maintenance that varies from state to state, which has witnessed a commercial revolution of hair products is now ready with a scientific journal dedicated to this important appendage of the body that is so deeply ingrained in dermatology. One wonders then why India did not make a mark internationally in scientific literature pertaining to hair. The reasons have been manifold—the lack of infrastructure, the lack of basic bench research, the struggle for survival with preoccupation with mundane things are amongst the reasons that explain why we have lagged behind. Hopefully, things will change and the journal will generate a deeper and a keener interest in hair, both normal and abnormal. I am happy to be on the editorial board of this fledgling journal and will help in generating more interest in it and wish it well always.

There are a few thoughts that have randomly crossed my mind very often while thinking about hair. They are jumbled, unmanaged and at random, just like unkempt and unmanageable hair! I take this opportunity to write those down taking advantage of the editorial board member's position that the association has bestowed upon me so kindly.

I hope the following common issues are dealt with as the association, its activities, its members, and its contributions in the journal grow. 'Hairs' are some starters, however!

Many of us have noted an association of low serum ferritin and diffuse hair loss in women. I have a substantial data linking low serum ferritin, often in single digits, with normal or marginally low hemoglobin and diffuse hair loss. A finding that was first documented in the United States caught the fancy of many of us. I feel more documentation is in order. A similar association has been noted by many of us between serum vitamin B12 and premature canities which has such a big social significance in our culture. Work and experience needs to be documented and published. That is a way of sharing and disseminating ideas, opinions, and experiences.

A vast majority of our women, especially those residing in rural areas have long hair that extends at least till mid to lower back. The cultural practice of combing vigorously is many generations old and is at times a social activity. Combing this long hair leads to loss of cuticle, may make the hair more unmanageable and may actually lead to fracture of hair shaft. Despite such a large number of women attending our hospitals and clinics, we have not studied this phenomenon. It is time we did it.

Massaging the hair with oil is a practice as old as the hills in this country. There are hundreds of brands of hair oil commercially available and being aggressively advertised in the media. Children, men, and women use hair oil as a part of their routine grooming. While this is taken for granted as a cultural practice one has to look more closely at the associated phenomena. Hair oil has many myths associated with it which are as deeply ingrained as the practice of oil application. This fuels the marketing of useless products making claims that deviate from truth, products that mislead and lead to an undue expense in buying products that sell little more than a lubricant and hope! Many of them are riding on the protective back of a powerful marketing animal, 'ayurvedic product' or 'natural product'. This is an unfortunate phenomenon leading to useless spending of millions of rupees on unscientific products. This should be a glaring example of imprudent spending on drugs and personal care products for those interested in pharmacoeconomics. A relative lack of mandate on cosmetic products and personal care products only adds to this unfortunate confusion. Estheticians and cosmetologists running beauty parlors have a wide array of hair treatments which have hardly any scientific backing. Women flock to them in the fond hope of acquiring hair that is seen only in glitzy advertisements seen on mass media. The same principle applies to hair care products like shampoos and cleansers who make blatant claims of growth, lasting glow, bounce and body and reversal of hair loss. I hope this association and the journal brings such issues to the forefront and acts in the name of science.

And finally to the issue of hair disorders, we need to train existing and younger dermatologists diagnosing and managing hair disorders. Persons on the editorial board will hopefully help us in that endeavor. The term 'trichology' has unfortunately gained a bad press because of its use by nondermatologists, often cosmetologists and estheticians. Call it 'hair disorders' or 'trichology' this is an uncharted route in the wide ocean of dermatology. We clearly need people who can claim true expertise and practical knowledge. India, with its overwhelming majority of dermatologists involved in private practice and who do not have mandatory relicensing or CME requirements will need these people to educate them in this area. Disorders of the hair that have until now been explained by practitioners as a result of poor nutrition, lack of grooming, hormonal imbalance, etc. need to be documented. Our brethren in the community will need to publish these entities more to educate the rest of us. I hope the interest in hair disorders is genuine and is not just commercially driven because of the revenue that it earns in procedures like hair restoration surgery.

I wish the organization and the journal well, today and always. For once I reap the benefits of being in the late 40s with Hamilton grade IV hair loss and the inevitable canities; this status gives me the much awaited seniority to implore the younger generation to take active interest in disorders of the hair. I look forward to learning more about this enigmatic appendage through my association with the *International Journal of Trichology* and the 'Hair Research Society of India'.

Here's to Hair!

